# Supplementary material for: Identification of photoperiod-induced specific miRNAs in the adrenal glands of Sunite sheep (Ovis aries)
Source: Front Vet Sci. 2022 Jul 22;9:888207. doi: 10.3389/fvets.2022.888207 (PMC9354845; doi:10.3389/fvets.2022.888207)
Supplement: Supplementary file 1 [file Data_Sheet_1.ZIP › Supplementary file/miRDeep2 file.zip!/miRDeep2 file/Novel_Structure/SP42A2/Novel_196.pdf]

Provisional ID	:	NC_019459.2_4436
Score total	:	2.6
Score for star read(s)	:	-1.3
Score for read counts	:	0
Score for mfe	:	2.3
Score for randfold	:	1.6
Score for cons. seed	:	
Total read count	:	198
Mature read count	:	198
Loop read count	:	0
Star read count	:	0

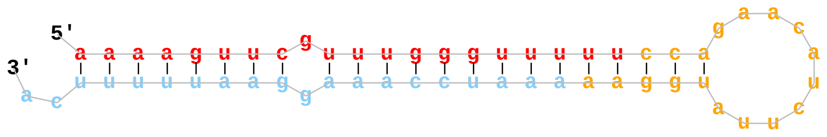

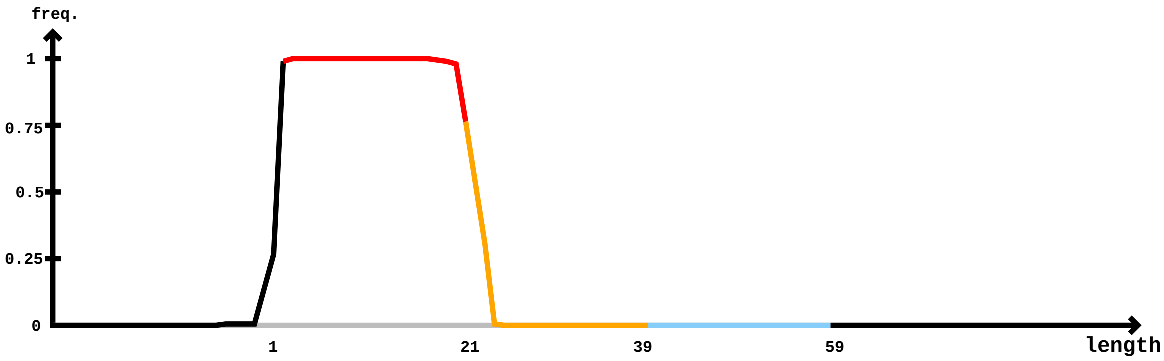

Mature Star

```
5'- ucaaauaauauuggguuggcugaaaaguucguuggguuuuuccagaacaucuuauggaaaaauccaaaggaauuuuucagccaacacaauauuaacuuucacagcuuugu  -3'   exp
    .....((((((((.((((((((((((((((.((((((((((((((..........)))))))))))))).)))))))))))))))).))))))))...............   reads   mm   sample
    ................uggcugaaaaguucAuuugggu........................................................................   1       1    seq
    ....................ugaaaaguucguuggguuuu......................................................................   7       0    seq
    ....................ugaaaaguucguuggguuuuu.....................................................................   17      0    seq
    ....................ugaaaaguucguuggguuuuuc....................................................................   2       0    seq
    .....................Aaaaaguucguuggguuuu......................................................................   23      1    seq
    .....................gaaaaguucguuggguuuu......................................................................   1       0    seq
    .....................gaaaaguucguuggguuuuu.....................................................................   2       0    seq
    ......................aaaaguucguuggguu........................................................................   1       0    seq
    ......................aaaaguucguuggguuu.......................................................................   2       0    seq
    ......................aaaaguucguuggguuuu......................................................................   12      0    seq
    ......................aaaaguucguuggguuuCu.....................................................................   1       1    seq
    ......................aaaaguucgCuuggguuuuu....................................................................   1       1    seq
    ......................aaaaguucguuggguuuuG.....................................................................   2       1    seq
    ......................aaaaguucguuggguuuuu.....................................................................   61      0    seq
    ......................aaaaguucAuuggguuuuuc....................................................................   1       1    seq
    ......................aaaaguucguuggguuuuuG....................................................................   1       1    seq
    ......................aaaaguucguuggguuuuuA....................................................................   3       1    seq
    ......................aaaaguucguuggguuuuucA...................................................................   57      1    seq
    ......................aaaaguucguuggguuuuucG...................................................................   1       1    seq
    ......................aaaaguucguuggguuuuucAa..................................................................   1       1    seq
    .......................Uaaguucguuggguuuuucc...................................................................   2       1    seq
```
